# The vital role of animal, marine, and microbial natural products against COVID-19

**DOI:** 10.1080/13880209.2022.2039215

**Published:** 2022-03-02

**Authors:** Aljawharah A. Alqathama, Rizwan Ahmad, Ruba B. Alsaedi, Raghad A. Alghamdi, Ekram H. Abkar, Rola H. Alrehaly, Ashraf N. Abdalla

**Affiliations:** a Department of Pharmacognosy, Faculty of Pharmacy, Umm Al-Qura University, Makkah, Saudi Arabia; b Department of Natural Products and Alternative Medicines, College of Clinical Pharmacy, Imam Abdulrahman Bin Faisal University, Dammam, Saudi Arabia; c Department of Pharmacology and Toxicology, Faculty of Pharmacy, Umm Al-Qura University, Makkah, Saudi Arabia

**Keywords:** SARS-CoV-2coronavirus, target, pandemic, microorganisms, combination treatment

## Abstract

**Context:**

Since the outbreak of SARS-CoV-2, researchers have been working on finding ways to prevent viral entry and pathogenesis. Drug development from naturally-sourced pharmacological constituents may be a fruitful approach to COVID-19 therapy.

**Objective:**

Most of the published literature has focussed on medicinal plants, while less attention has been given to biodiverse sources such as animal, marine, and microbial products. This review focuses on highlighting natural products and their derivatives that have been evaluated for antiviral, anti-inflammatory, and immunomodulatory properties.

**Methods:**

We searched electronic databases such as PubMed, Scopus, Science Direct and Springer Link to gather raw data from publications up to March 2021, using terms such as ‘natural products’, marine, micro-organism, and animal, COVID-19. We extracted a number of documented clinical trials of products that were tested *in silico, in vitro*, and *in vivo* which paid specific attention to chemical profiles and mechanisms of action.

**Results:**

Various classes of flavonoids, 2 polyphenols, peptides and tannins were found, which exhibit inhibitory properties against viral and host proteins, including 3CLpro, PLpro, S, hACE2, and NF-κB, many of which are in different phases of clinical trials.

**Discussion and conclusions:**

The synergistic effects of logical combinations with different mechanisms of action emphasizes their value in COVID19 management, such as iota carrageenan nasal spray, ermectin oral drops, omega-3 supplementation, and a quadruple treatment of zinc, quercetin, bromelain, and vitamin C. Though *in vivo* efficacy of these compounds has yet to be established, these bioproducts are potentially useful in counteracting the effects of SARS-CoV-2.

## Introduction

The recent outbreak of the novel coronavirus (COVID-19) began in December 2019, in Wuhan city, China, when several cases of pneumonia of an unknown origin were reported. The virus responsible was identified as SARS-CoV-2 (severe acute respiratory syndrome coronavirus 2) and was epidemiologically traced back to the Huanan seafood and wet animal wholesale market in Wuhan. The outbreak of COVID-19 quickly escalated to epidemic proportions throughout China and began rapidly spreading to other countries until WHO declared it a global pandemic in March 2020 (Sharma et al. 2020).

The last two decades have seen the emergence of several newly identified coronaviruses, such as Middle Eastern respiratory syndrome coronavirus (MERS-CoV) in Saudi Arabia, haemorrhagic fever viruses (Lassa, Ebola) in West Africa, and novel coronaviruses including severe acute respiratory syndrome coronavirus (SARS-CoV) resulting a significant mortality and economic loss. Virus pandemics have caused a tremendous number of deaths and it is therefore critical to prevent the spread of emerging viruses (Sharma et al. 2020).

*Coronavirus* is a positive-sense, single-stranded RNA virus (diameter of 60–140 nm) belonging to the Coronaviridae family which together with Roniviridae and Arteriviridae, belongs to the Nidovirales order. The subfamilies under Coronaviridae are the Torovirinae and Coronavirinae subfamilies; the latter are further subclassified into α-, β-, γ-, and δ-COVs with SARS-CoV-2 belonging to the β-COV group. This RNA virus family is known for its diversity in different animal species as well as its ability to attack different body systems such as the respiratory, hepatic, nervous system, and gastrointestinal systems. The term coronavirus comes from the crown-like (‘corona’ from the Latin) appearance of the club-like projections of spike glycoproteins on the surface envelope of the virus, which can be perceived with an electron microscope (Hassan et al. 2020). SARS-CoV-2 was first discovered at an animal market in China and can be transmitted from animals to humans. Human-to-human transmission is common if the individual is in the contagious phase of the infection, either symptomatically or asymptomatically. Common paths of transmission are via airborne droplets entering the nose, mouth or eyes, as well as contact with surfaces such as skin or faecal matter. Long-range transmission has also been detected from inhalation of airborne dust (Sharma et al. 2020).

The primary site of infection is the respiratory system, where it results in flu-like symptoms with an incubation period of 2–14 days. Fever, cough, fatigue, slight dyspnoea, sore throat, headache, conjunctivitis, and gastrointestinal issues are common symptoms with progression to severe cases of breathing difficulties. The virus may produce respiratory distress syndrome, respiratory failure, and systemic inflammation. In high-risk individuals the virus has been shown to cause sepsis, affect cardiovascular functioning, attack the heart and other organs. A high mortality rate has been observed in age above 60 and individuals with comorbidity conditions (Pascarella et al. 2020). Per available figures, COVID-19 has currently affected more than 200 countries (Sharma et al. 2020). Until beginning of July 2021, more than 186 million people have been diagnosed with COVID-19 and more than 4 million have died so far (WHO 2021).

## The genome organization, viral proteins and life cycle of SARS-CoV-2

### Genome organization

Based on the available virological data, the similarity between SARS-CoV-2 and SARS-CoV is about 70% and it shows around 95% homology with bat coronavirus. Its viral RNA genome ranges in length from 26 to 32 kilobases, having a variable number of open reading frames (ORFs), with a unique replication strategy. Furthermore, translation of the replicase polyproteins is made possible by the genome’s 5′ cap structure and 3′ poly (A) tail which gives it the capacity to behave as messenger RNA (Hassan et al. 2020).

The 5′ cap end of the viral genome has a leader series and untranslated region (UTR) composed of multiple regions. These are crucial to the formation of the many stem loop structures that are necessary for RNA replication and transcription. At the accent gene there are transcriptional regulatory sequences (TRSs) composed of a specific portion of 50–100 nucleotides required for the expression of each of those genes. The RNA structures needed to replicate and synthesize RNA are located in the 3′ UTR. The two-third (20 kilobases) of the genome consists of replicase genes known as open reading frames 1a and ab (ORF1ab), and encoded non-structural proteins (nsp), whereas the remaining region of the total viral genome (10 kilobases) encodes structural and accent proteins such as structural proteins involving spike (S), envelope (E), membrane (M), and nucleocapsid (N) proteins. Furthermore, the structural genes such as ORF3a, ORF3d, ORF6, ORF7a, ORF7b, ORF8, ORF9b, ORF14, and ORF10 genes encode nine accessory proteins. The CoV genome is structured in the following order: 5′-leader-UTR-ORF-S-E-M-N-accessory proteins genome-3′ UTR-poly (A) tail with accessory genes interspersed among the structural genes at the 3′ end of the genome (Pal et al. 2020; Yadav et al. 2021).

### Viral proteins

**Figure 1.**
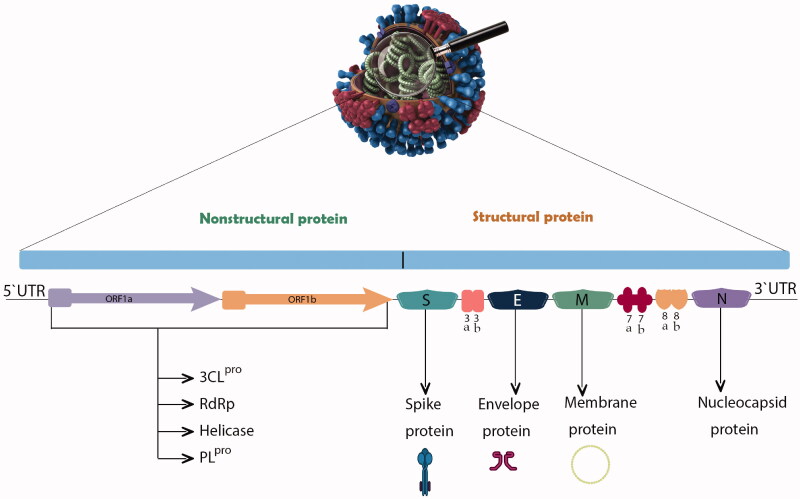
Genome organization of SARS-CoV-2 sequence and the various proteins encoded by different genes.

SARS-CoV-2 has four important structural proteins: S, M, E and N proteins. There are also several non-structural and/or accessory proteins which are able to alter the structure and functions of the virus (Figure 1) (Yadav et al. 2021). The S glycoprotein belongs to the type I transmembrane N-linked glycosylated protein family and comprises 1,273 amino acids; it is thus the main target of neutralization antibodies (Ou et al. 2020). This S glycoprotein is assembled in trimeric associated polypeptide chains and each monomer (180 kDa) has two subunits, S1 and S2, to mediate membrane fusion and virus entry with different domains as follows, from the N-terminal domain (NTD), receptor binding motif having receptor binding domain (RBD), furin cleavage site (S1/S2), fusion peptide (FP), central helix (CH), connecting domain (CD), heptad repeat (HR1/2), transmembrane domain (TM), and cytoplasmic tail (CT). Through the RBD-S1 down-to-up conformational transition, virus interaction with an entry receptor for SARS-CoV-2 named human angiotensin-converting enzyme 2 (hACE2) is facilitated, and hence, cell recognition and binding take place (Duan et al. 2020; Ou et al. 2020; Yadav et al. 2021). S glycoprotein is activated by one of the host proteases specifically at the S2′ site at the FP site, which is essential for membrane fusion activity (Saxena et al. 2020). A different feature of the S protein is its NTD-linked glycosylation, which has a role in conformation-dependent dynamic changes as it covers mostly the surface area (Yadav et al. 2021).

The N protein is a 419-amino acid structural protein with three structural domains: an N terminal region, an RNA-binding domain (linker region) and a C terminal region, thus allowing it to orchestrate RNA binding. It is highly expressed during infection and restricts host immune response, and is therefore a good target for vaccine development. It also participates in RNA packaging, organization of the viral genome, virion assembly, enhancement of virus transcription efficiency, intracellular protein transport, DNA degradation and interference in host translation (Gao et al. 2021; Yadav et al. 2021).

The M protein is an O-linked glycoprotein composed of 222 amino acids that are considered to be the most abundant structural proteins. It has three transmembrane domains, the N terminal domain and cytoplasmic domain inside the virion. It interacts with other structural proteins such as with the N protein to facilitate its stabilization as N protein-RNA complex in the virion, encapsulation of the RNA genome, thus promoting completion of viral assembly. It also interacts with the S protein and may affect the entry of the virus into the host as well as host cell attachment (Thomas 2020).

The E protein is an integral membrane protein (76–109 amino acids) with three distinctive domains, the N terminal, the hydrophobic, and the C terminal domains. As it is hydrophobic and forms viroporins, it mediates viral assembly on its release. Also, its heterotypical interaction with nsps, such as nsp2 and nsp3, is crucial curvature in the endoplasmic reticulum membrane. Its tail is partly embedded in the cytoplasm and targets the *cis*-Golgi complex region, while the N terminal has additional Golgi complex. The virion exits by the E protein when the ionic gradient is levels out in the endoplasmic reticulum Golgi intermediate compartment (ERGIC) and Golgi compartment. Thus, this protein has an essential role in viral pathogenesis, virion release and assembly (Satarker and Nampoothiri 2020).

The ORF1ab region is translated into 2 polyproteins, pp1a corresponding to nsp1–nsp11, and pp1ab compromising nsp12-nsp16; these make up the replicase/transcriptase complex (RTC). RTC has several enzymes that play a major role in viral replication process, such as papain-like protease; PL^pro^ (nsp3), main protease; 3CL^pro^; M^pro^ (nsp5), nsp7-nsp8 primase complex, primary RNA-dependent RNA polymerase (RdRp; nsp12), helicase/triphosphatase (nsp13), exoribonuclease (nsp14) and endonuclease (nsp15) (Alsobaie 2021).

The accessory proteins belong to another class of proteins encoded in SARS-CoV-2 and are less well-known compared to the rest. There are two major reasons for this; first, they are not essential or part of viral structure/replication, however, they play a role in viral spread and pathogenicity. Second, predicting protein complexity by bioinformatics is challenging due to their complex nature as short and overlapping ORFs (Michel et al. 2020). Five ORFs encoding accessory genes (ORF3a, ORF6, ORF7a, ORF7b, and ORF8) encode nine accessory proteins including ORF3a, 3d, 6, 7a, 7b, 8, 9b, 14, and 10 and N gene (ORF9b and 14) encodes novel overlapping ORF3d (earlier known as 3b) (Yadav et al. 2021).

### Viral life cycle

The SARS-CoV-2 life cycle is composed of early and late events of different stages; (1) attachment to host cell surface; (2) viral penetration and uncoating; (3) replication and transcription and (4) viral assembly and release. Initial attachment of the virion to the host cell is via its receptor RBD-S1 subunit (with down-to-up conformation) binding to hACE2, that primarily exists in epithelial cells of the lungs and small intestine, kidney, heart, and other tissues. Following this binding, the S protein is cleaved between the boundary of the S1 and S2 via protease-mediated cleavage at the S1/S2 cleavage site, thus triggering irreversible membrane fusion (Yadav et al. 2021). In addition, the endocytosis of S protein containing virus is a pH dependent process where acidic endosomal pH leads to activation of endocytosed virions and viral accessibility to the host cytoplasm. The endosome is digested by enzymatic reaction to release the viral genome (Haque et al. 2020). Viral replication cycle now begins where viral RNA serves as functional mRNA to be translated. ORFs facilitate RNA attachment to the host cell’s ribosome, which enables the viral replicase gene (ORF1ab) to be translated, resulting in the synthesis of the two main polyproteins; pp1a and pp1b. Both go through autoproteolytic activation, giving 16 nsps, which together form the RTC, resulting in copies of the viral RNA being synthesized many times over. After producing a multiple 50-nested set of negative sense RNAs and a 30-nested set of positive sense, associating with the host ribosome leads to the synthesis of structural and accessory proteins to build the viral structure (Haque et al. 2020; Yadav et al. 2021). Furthermore, those proteins are either translated by endoplasmic reticulum-bound ribosomes or exist freely in the cytosolic ribosomes of the host cell. Later viral assembly takes place at the ERGIC, with transportation in vesicles via a secretory pathway and elimination from the host cell through exocytosis in order to spread to different parts of the body (Al-Horani et al. 2020; Yadav et al. 2021).

## The history of marine-animal-microbes as medicine for infectious diseases

**Figure 2.**
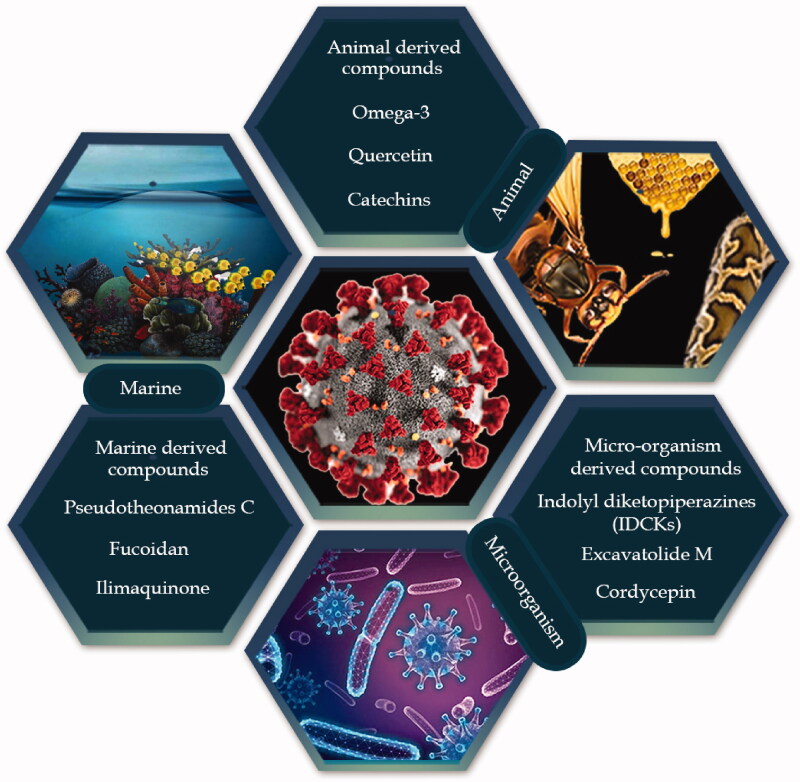
Natural-products-derived from various sources to combat COVID-19 infection.

Infectious diseases have affected human life since hunter-gatherer times and in the last century this field has witnessed a revolution due to the development of antibiotic-based therapy (Watson et al. 2008). Infectious diseases such as malaria, tuberculosis and smallpox have been a central challenge for public health throughout the history of medicine. As infectious diseases started to spread geographically, communities began to explore the potential of their indigenous flora to yield therapeutic preparations, and after extensive empirical experiment plants were identified that showed activity against infection and disease (Wabo Poné et al. 2011). The plant-world is not the only source of remedies to fight infections; animal products have also been used since the origin of mankind. For instance, the products of honey from *Apis mellifera* (L.), (Apidae) and snake venom have long been used in traditional medicinal systems for the treatment of ailments caused by microorganisms (Figure 2) (Mandal and Mandal 2011).

Microbes are also a source of anti-infectious agents and arguably the most important breakthrough in the history of medicine was the discovery of penicillin, when Alexander Fleming noticed the absence of visible staphylococci near an area of fungal growth, and subsequently won the Nobel prize for the discovery of antibiotic activity (Ligon 2004). Other successful examples of anti-infectious agents from fungi are strobilurins and oudemansin belonging to of the class of fungal polyketides and were first used to protect agricultural crops from fungal diseases. Their development arose when two agaricomycetes *Strobilurus tenacellus* (Pers) Singer and *Oudemansiella mucida* (Schrd.) Hoehn. (Physalacriaceae), were found to resist fungal infection (Jakubczyk and Dussart 2020).

Marine environments are a rich source of a broad variety of living organisms such as sponges, tunicates, fish, soft corals, nudibranchs, sea hares, weeds, algae, bryozoans, prawns, shells, sea slugs, and marine microorganisms with an established role as anti-infective agents. The importance of the marine environment arises from the fact that 80% of plant and animal species in the world exist in oceans. A large number of marine bacteria appear to be promising anti-infectious agents. A study investigated five bacterial strains from four species of marine sponge and 21% of the isolates demonstrated good antibacterial activity. A common example is cephalosporin C, obtained from the marine fungus, *Cephalosporium acremonium* Corda (Cephalothecaceae). The extract from the Tunisian sponge *Sarcotragus* sp. (Irciniidae), also revealed an anti-leishmanial activity with morphological alterations in promastigotes of leishmania. Likewise, isonitrile has been isolated from a Japanese sponge named *Acanthella* sp. (Dictyonellidae) which showed anti-malarial, anti-antifungal, anthelmintic, and antifouling activities. An anti-herpes simplex virus-1 agent has been developed from exo-polysaccharides extracted from a French marine sponge named *Celtodoryx girardae* (Perez, Perrin, Carteron, Vacelet, & Boury-Esnault, Coelosphaeridae) (Malve 2016).

## Materials and methods

All relevant information about chemical class, pharmacological effects, and assessment methods of natural products from non-plant sources were collected from the available literature. Electronic databases such as PubMed, Scopus, Science Direct, Springer Link, Web of Science and Google Scholar were used to gather raw data from publications up to March 2021. The terms searched were natural products, natural compounds, marine, micro-organism, and animal, along with terms corresponding to COVID-19, SARS-CoV-2 and coronavirus. Chemical structures were drawn using ChemBioDraw Ultra 14.0 software. The PubChem database was used to check the IUPAC names of compounds from natural sources. Compounds that had been isolated from marine, animal, and micro-organism sources, and which had antiviral and known therapeutic action against SARS- CoV-2, in either *in silico*, *in vitro,* or clinical studies, were noted as potential candidates for further research.

In searching extensively for publications to discuss in the literature review the search criteria included any journal article in the English language containing the words COVID-19, SARS-CoV-2 or coronavirus, along with preclinical and clinical studies of compounds from marine, animal, and micro-organism sources. The initial search was followed by filtration of the relevant data for review. At the end of the search, single compounds, either alone or in combination, which had been proven to have anti-viral activity, were assessed for their potential contribution to COVID-19 management.

## Natural products investigated as potential drug candidates for COVID-19 treatment

**Figure 3.**
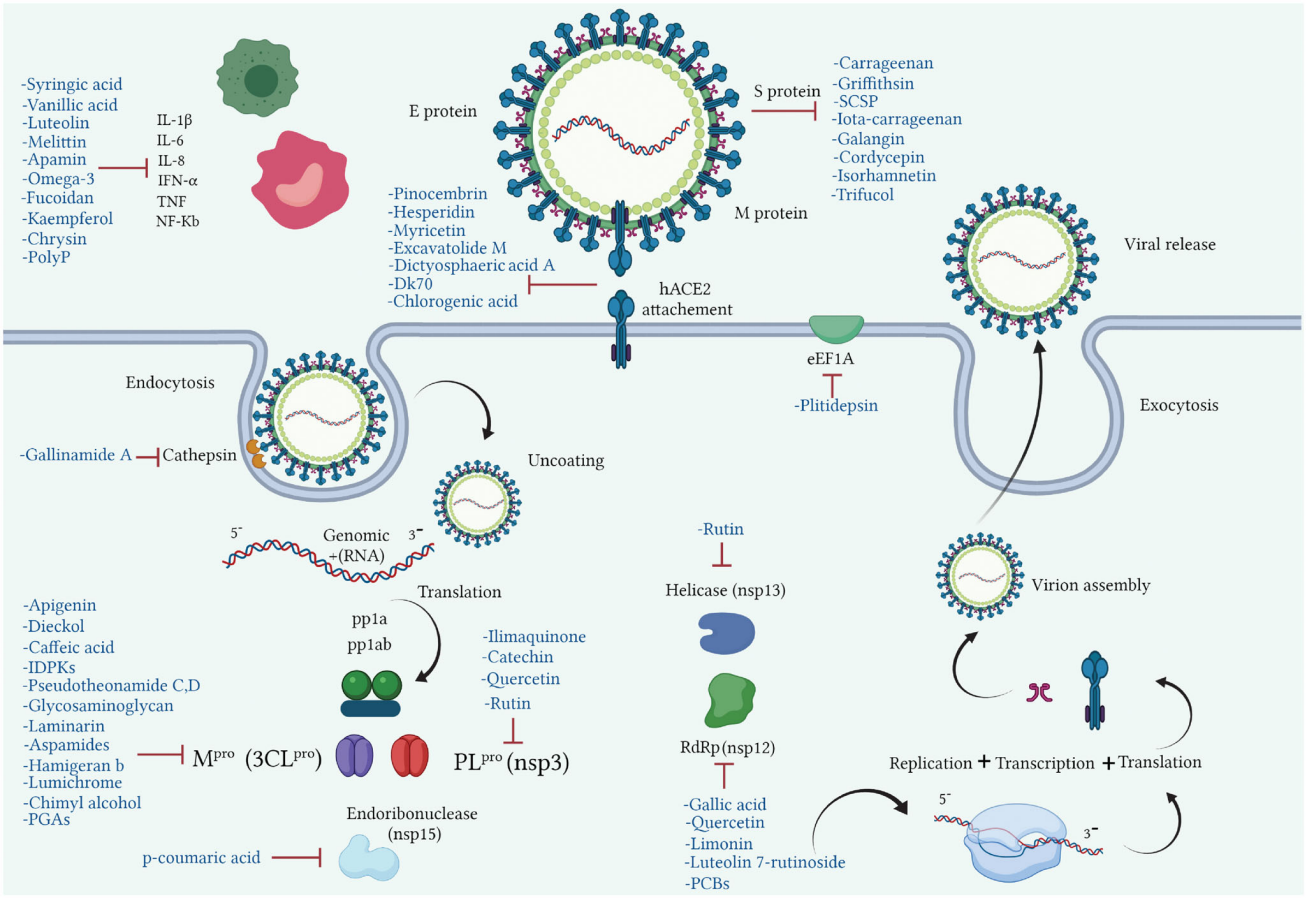
Molecular targets of anti-viral natural products from marine, animal and microbial sources.

COVID-19 is similar to other infectious diseases that have caused pandemics in the last century and which have required effective treatment and prevention strategies. Unfortunately, there is no treatment as yet for the SARS-CoV-2 virus that would eliminate this disease, thus the search for effective agents continues. Natural products from plant, animal, marine, and microbial sources have been extensively explored over the years and used as platforms in drug development and discovery programs. Most of the existing literature on this subject focuses on natural products from plants, however, much less attention has been given to those isolated from unexplored natural sources such as marine, animal and microbial. There are many of these that are already known for their unique chemical profiles, which are accompanied by interesting pharmacological activities related to the different mechanisms used by each phytochemical class capable of exhibiting biological activity (Figure 3).

During the current coronavirus pandemic, natural compounds, marine, micro-organism, and animal, have served as milestones in the search for new or repurposed naturally-derived drugs to develop effective anti-viral lead molecules. Chemical classes of compounds such as flavonoids, alkaloids, phenols, lignans, tannins and peptides which have marine, animal or microorganism origins demonstrate different inhibitory actions on SARS-CoV-2. Such activity has been investigated using different analytical approaches such as *in silico*, *in vitro,* and clinical studies with various effect on virus pathogenesis stages such as viral entry and replication, structural and non-structural proteins, inflammatory and immunostimulatory effects (Table 1).

**Table 1.** Anti-SARS-CoV-2 natural compounds with different chemical profiles tested in different assessment methods. NI: not identified. The papain-like protease (PL^pro^).

Name	Source	Structure	Target	IC_50_ value	Reference
Apamin	Bee venom *A. mellifera*	C_79_H_131_N_31_O_24_S_4_	IKK, TNF-α IL-1β, IL-6, NF-κB pathway and MMP-9	NI	(Lin and Hsieh 2020)
Apigenin	Honey and propolis	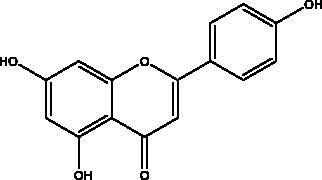	S protein, 3CL^pro^	280.8 μM	(Ryu et al. 2010)
Bradykinin-potentiating peptide	Snake venom	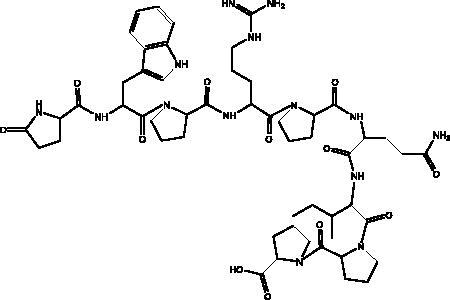	hACE2, NO	NI	(Gouda and Mégarbane 2021)
Caffeic acid phenethyl ester	Propolis	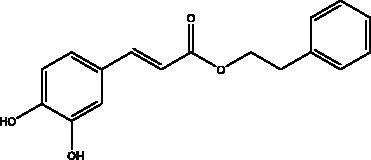	3CL^pro^	–5.68 Kcal/mol	(Kumar et al. 2021)
Catechins	Honey, propolis, and royal jelly	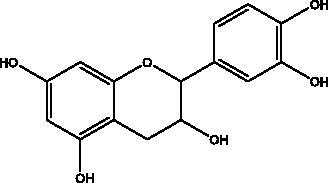	3CL^pro^, cathepsin L, RBD of S protein, nsp6, PL^pro^ , ionophores for zinc ions, IL-1β and IL-6 signalling pathways	–73 ± 2 μM (3CL^pro^) -Range (–7.59 to –37.39 kcal/mol) (cathepsin L, RBD of S protein, nsp6)	(Chourasia et al. 2021; Mhatre et al. 2021; Mishra et al. 2021)
Chimyl alcohol	*Desmapsamma anchorata* sponge	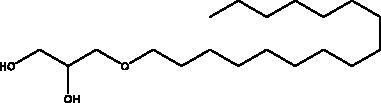	M^pro^	NI	(Hamoda et al. 2021)
Chlorogenic acid	Propolis	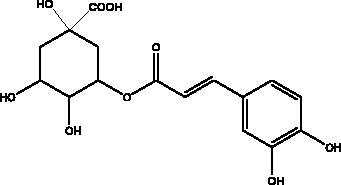	RBD domain, S protein, hACE2	–0.87 kcal/mol	(Yu et al. 2020)
Chrysin	Honey, bee pollen and royal jelly	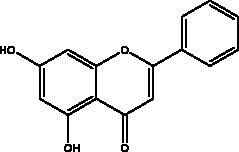	S protein, 3CL^pro,^ NF-кB signalling pathway	400 μM	(Abedi et al. 2021)
Cordycepin	*Cordyceps militaris* fungus	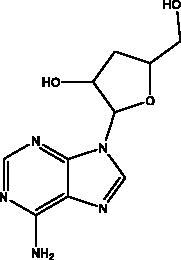	S protein, M^pro^	NI	(Verma 2020)
p-coumaric acid	Honey, propolis and bee pollen	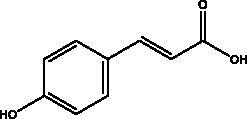	S protein, HSPA5, endoribonuclease	5.63 kcal/mol	(Elfiky 2021)
Dictyosphaeric acid A	Green alga Dictyosphaeria versluyii	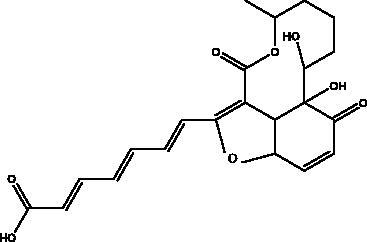	TMPRSS2-S PPIs, hACE2-S	NI	(Senapati et al. 2021)
Dieckol and eckol	Brown algae *Ecklonia cava*	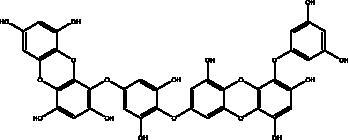 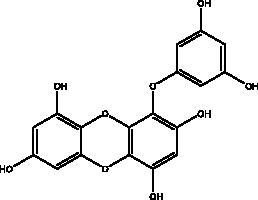	3 CL^pro^	Dieckol: –11.51 kcal/mol, Eckol: –8.19 kcal/mol	(Arunkumar et al. 2021)
Esculetin-4-carboxylic acid methyl ester	Marine sponge *Axinella cf. corrugata*	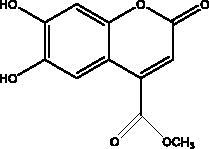	M^pro^	46 μM 112 μM (depends on used assay)	(Coelho et al. 2020; Hamoda et al. 2021)
Excavatolide M	Soft coral *Formosan gorgonian Briareum*	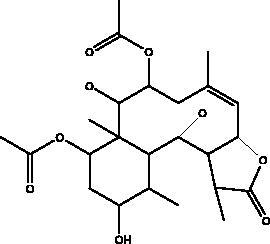	TMPRSS2-S PPIs, hACE2-S	NI	(Rahman et al. 2020)
Galangin	Honey	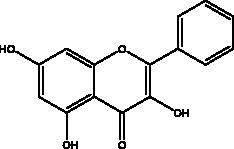	S protein	–8.2 Kcal/mol	(Jain et al. 2021)
Gallic acid	Honey	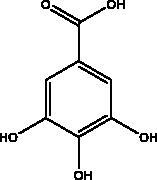	RdRp	–7.55 kcal/mol	(Abdo et al. 2021)
Gallinamide A	Marine cyanobacterial	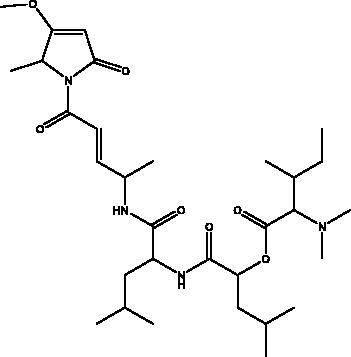	Cathepsin	IC_90_ of 88 nM in	(Ashhurst et al. 2020)
Glycosaminoglycan	Marine bacteria, Pseudomonas	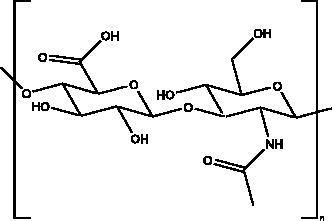	M^pro^	–7.98 kcal/mol	(Vijayaraj et al. 2020)
Griffithsin	Red alga *Griffithsia* sp	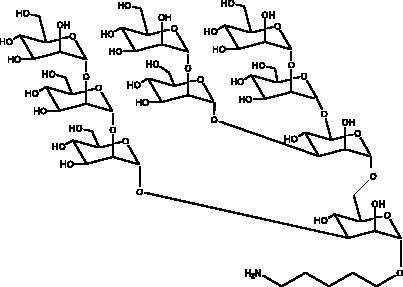	S protein	293 nmol/L 3 nmol/L 323 nmol/L (depends on used assay)	(Cai et al. 2020)
Fucoidan	Brown seaweeds (Phaeophyceae)	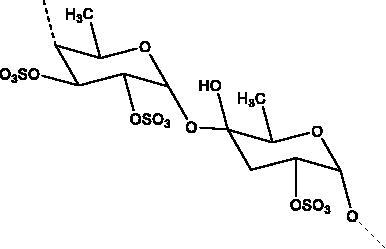	S protein	15.6 μg/mL	(Fitton et al. 2020)
Hamigeran b	Marine sponge, *Hamigera tarangaensis*	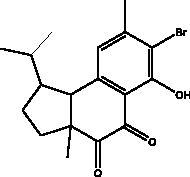	3CL^pro^	7.98 kcal/mol	(Vijayaraj et al. 2020)
Hesperetin	Honey and royal jelly	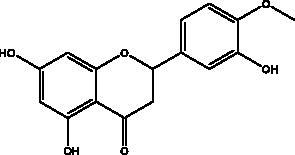	hACE2, 3CL^pro^	–7.45 kcal/mol (hACE2) 8.3 μM (3CL^pro^) 60 μM (3CL^pro^)	(Guler et al. 2021)
Ilimaquinone	marine sponge *Hippospongia metachromia*	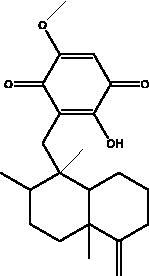	PL^pro^, 3CL^pro^, nsp10, nsp14	–8.1 kcal/mol (PL^pro^) –7.1 kcal/mol (3CL^pro^) –7.6 kcal/mol (nsp10) –8.1 kcal/mol (nsp14)	(Surti et al. 2020)
Indolyl diketopiperazines (IDPKs)	Fungi Aspergillus versicolour	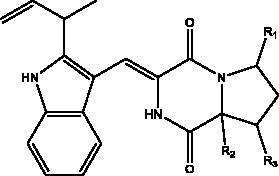 R1 = α-OCH_2_CH_3_ R2 = α-H R3 = H aspamides A R1 = β-OCH_2_CH_3_ R2 = α-H R3 = H aspamides B R1 = H R2 = OH R3 = H brevianamide Q 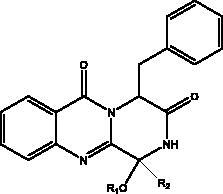 R1 = H R2 = CH_3_ aspamides F R1 = H R2 = CH_2_CH_3_ aspamides G R1 = H R2 = H brevianamide M	3CL^pro^	Aspamides A: –5.389 Aspamides B: –4.772, Aspamides F: –5.146 Aspamides G: –4.962 Brevianamide M: –5.158 Kcal/mol	(Ding et al. 2020)
Inorganic polyphosphate (polyP)	Marine sponges, bacteria (*Cyanobacterium synepchcoccus*) t	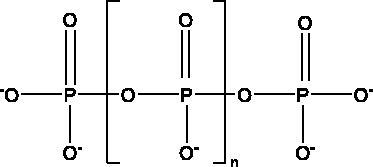	hACE2, RBD, NF-κB	1 μg/mL to 100 μg/mL	(Neufurth et al. 2020)
Iota-carrageenan	Red algae (Rhodophyta)	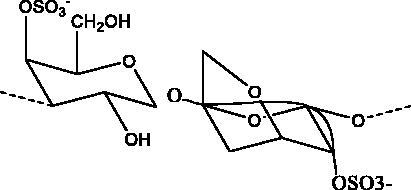	S protein	2.6 μg/mL	(Morokutti-Kurz et al. 2021)
Isorhamnetin	Propolis, bee pollen and royal jelly	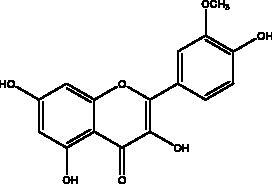	S protein, hACE2	2.51 μM	(Zhan et al. 2021)
					
					
Kaempferol	Honey, bee pollen, royal jelly	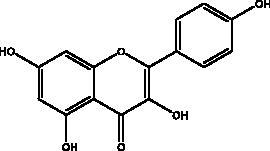	3a channel, TMPRSS2, IL-6, TNF-α, VEGF	–9.41 kcal/mol	(Berretta et al. 2020)
Limonin	Propolis	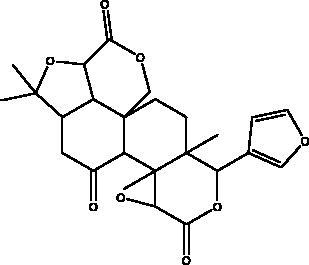	RdRp, hACE2	9 to –7.1 kcal/mol	(Berretta et al. 2020)
Lumichrome	Honey and propolis	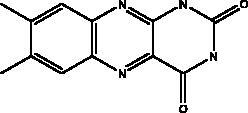	3CL^pro^	–5.205 Kcal/mol	(Hashem 2020)
Luteolin	Honey and propolis	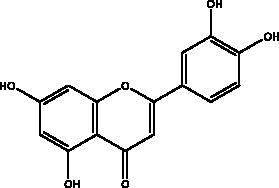	NF-κB	NI	(Khalil and Tazeddinova 2020)
Luteolin 7-rutinoside	Propolis	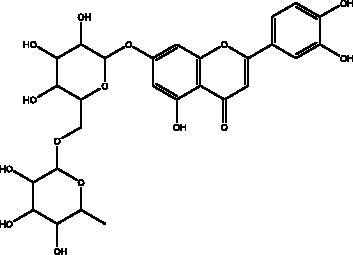	RdRp	–9.8 kcal/mol	(Alamri et al. 2020)
Melittin	Bee venom *A. mellifera*	C_131_H_229_N_39_O_31_	IKK, TNF-α IL-1β, IL-6, NF-κB pathway, MMP-9	NI	(Lin and Hsieh 2020)
Myricetin	Honey and propolis	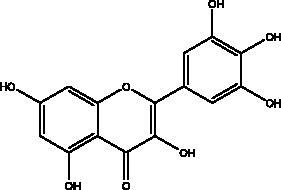	hACE2	–7.59 kcal/mol	(Guler et al. 2021)
Omega-3 fatty acids	Fish, other seafoods and algeas	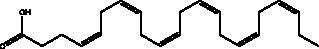 DHA 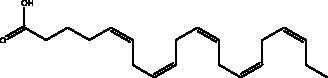 EPA	IL-1β, IL-TNF-α, COX, LOX pathway	NI	(Hathaway et al. 2020)
3‐Phenyllactic acid	Honeybee and propolis	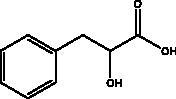	3CL^pro^	–5.867 Kcal/mol	(Hashem 2020)
Phycocyanobilins (PCBs)	Cyanobacteria, algae rhodophytes	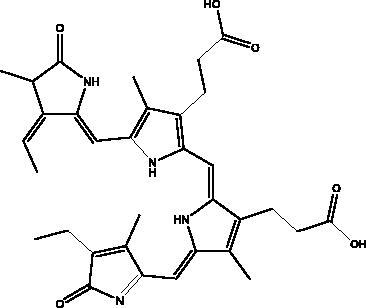	RdRp, M^pro^, S protein	–7.2 kcal/mol	(Geahchan et al. 2021)
Pinocembrin	Honey, propolis and royal jelly	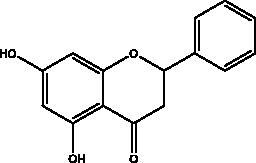	hACE2	–7.16 kcal/mol	(Guler et al. 2021)
Plitidepsin	Tunicate *Aplidium albicans*	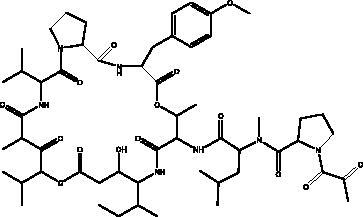	eEF1A	IC_90_ of 0.88 nM	(Taglialatela-Scafati 2021)
Polycyclic guanidine alkaloids (PGAs) (Rambescidin 786, Crambescidin 826)	Marine Poeciloscleridsponges	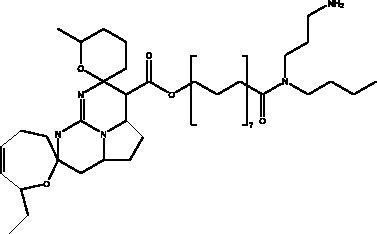	M^pro^, nucleocapsid phosphoprotein, nsp10	Ranhe from –9.06–6.49 kcal/mol)	(El-Demerdash et al. 2021)
Pseudotheonamides C and D	Marine sponge *Theonella swinhoei*	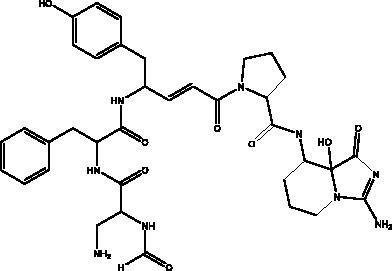 Pseudotheonamides C 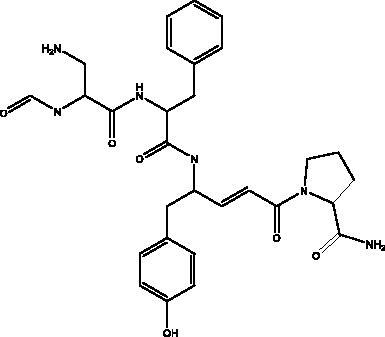 Pseudotheonamides D	M^pro^	Pseudotheonamides C: 11.6 kcal/mol Pseudotheonamides D: –10.7 kcal/mol	(Gentile et al. 2020)
Quercetin	Honey, propolis, and royal jelly	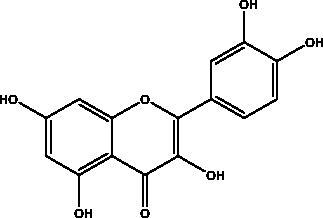	3CL^pro^, PL^pro^, RdRp, S protein hACE2, COX2, iNOS, NF-κB, ionophores for zinc ions	73 μM (3CL^pro^) 4.48 μM (hACE2) –40.9 (PL^pro^) – 5.41 kcal/mol (RdRp) 83.4 µM (S protein)	(Kandeel et al. 2020; Saakre et al. 2021)
Rutin	Propolis and bee pollen	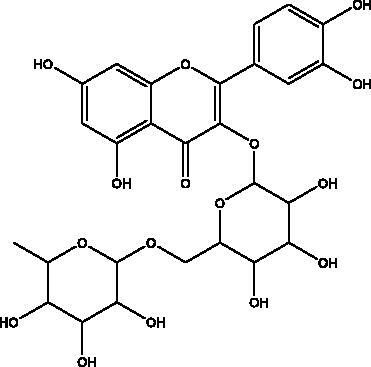	3CL^pro^ helicase, RdRp, PL^pro^, hACE2, TLRs	–9.2 kcal/mol (3CL^pro^) –6.9 kcal/mol (hACE2) Range –5.29 to –9.76 kcal/mol) (TLRs)	(Hu et al. 2020; Rehman et al. 2020; Agrawal et al. 2021)
Syringic acid	Honey	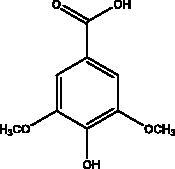	IL-4, IL-5, IL-13, and TNF-α ROS	NI	(Li et al. 2019)
Trifucol	Brown alga *Himanthalia elongate*	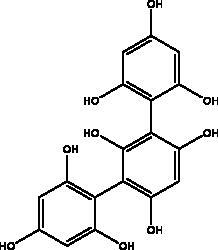	S protein, 3CL^pro^	–7.5 kcal/mol (S protein) –6.3 kcal/mol (3CL^pro^)	(Arunkumar et al. 2021)
Vanillic acid	Honey and propolis	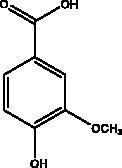	NFκB pathway	NI	(Khalil and Tazeddinova 2020)

### The S protein-hACE2 interaction

Several natural compounds have been shown to interfere with viral attachment through the interaction between hACE2 and the S protein. A lectin isolated from the red alga *Griffithsia* sp. (Wrangeliaceae), griffithsin, has demonstrated broad anti-viral activity. It significantly inhibits SARS-CoV-2 infection in a dose-dependent manner in a SARS-CoV-2 pseudovirus infection assay with the half maximal inhibitory concentration (IC_50_) = 293 nmol/L. The anti-viral activity against live SARS-CoV-2 infection was confirmed by immunofluorescence and qRT-PCR assay, with activity (IC_50_ = 3 nmol/L) that was eleven times stronger than that of one of the potent anti-virals for COVID-19, remdesivir. More specifically, it inhibited SARS-CoV-2 viral adhesion *in vitro* through S-mediated cell–cell fusion and S1 subunit and RBD with an IC_50_ of 323 nmol/L by targeting the glycosylation sites in S1 subunit (Cai et al. 2020). The fungal decalactone dictyosphaeric acid A, obtained from the green alga *Dictyosphaeria versluyii* (Weber-van Bosse, Siphonocladaceae), has shown its inhibitory properties against disrupt TMPRSS2-S PPIs, a host protein that facilitates viral entry, and hACE2-S (Rahman et al. 2020; Senapati et al. 2021).

Marine sulphated polysaccharides that have been extracted from brown and red algae have been proven to interfere with viral adhesion to host cells and these include sea cucumber sulphated polysaccharide (SCSP) extracted from fresh sea cucumbers *Stichopus japonicus*, (Selenka, Holothuroidea), fucoidan in the cell walls of the brown seaweeds (Phaeophyceae) and iota-carrageenan from red algae (Rhodophyta). SCSP and fucoidan were found in different concentrations to significantly slow down SARS-CoV-2 infection in Vero E6 cells, with IC_50_ of 9.10 and 15.6 μg/mL respectively. The structural relationship activity indicates the importance of the degree of sulphation in which SCSP with the highest sulphation degree showed the best inhibitory effect. SCSP was shown to effectively slow the growth of infection by a pseudotype virus, indicating its interaction with S protein (Song et al. 2020). Furthermore, fucoidan led to reduced injury to animal lungs caused by viral infection (Fitton et al. 2020). With an IC_50_ value of 2.6 μg/mL, iota-carrageenan neutralized spike pseudotyped lentivirus particles to prevent viral entry (Morokutti-Kurz et al. 2021). Cordycepin is a bioactive metabolite of fungus called *Cordyceps militaris* ((L.) Fr., Clavicipitaceae), which has demonstrated *in silico* properties with strong binding affinity with SARS-CoV-2 S protein (–145.3) and M^pro^ (–180.5) (Verma 2020). Furthermore, phospholipases A2 (PLA2s) obtained from snake venom *Bothrops jararaca* (Wied-Neuwied, Viperidae) that hydrolysis of phospholipids at a specific position protect Vero E6 cells at various degrees from SARS-CoV-2 with IC_50_ values ranging from 0.06 to 7.71 μg/mL. PLA2s have been found to suppress viral entry through S protein inhibition mediated by cell-cell fusion (Siniavin et al. 2021). In addition, another peptide derived from snake venom, bradykinin-potentiating peptide (BPP-10c), is known to be a short proline-rich peptide with remarkable functional variabilities. BPP-10c strongly inhibited hACE2, and thus viral entry, as well as having an effect on the inflammatory response of host cells and anti-oxidants through NO mediated effect (Gouda and Mégarbane 2021). Inorganic polyphosphate (polyP) obtained from marine sponges, bacteria *Cyanobacterium synepchcoccus,* (Nägeli, Cyanobacteriaceae) that shown to bind the S protein, inhibiting its interaction with hACE2 and thus inhibiting viral entry. In addition, hACE2 inhibitor screening assays reveal the disintegration of hACE2 on host cells at concentrations of 1–100 μg/mL in RBD (SARS-CoV-2). This bacterium also suppresses the NF-kB pathway and reduces the cytokine storm typically associated with COVID-19 infection. Furthermore, it has synergistic anti-viral effects when combined with anti-inflammatory compounds such as dexamethasone and anti-oxidant compounds such as quercetin (Neufurth et al. 2020; Geahchan et al. 2021).

Another chemical class showing promise in inhibiting viral attachment is that of phenolic compounds, such as *p*-coumaric acid, obtained from honey, which is reportedly an inhibitor of S protein. Furthermore, it binds to cell-surface HSPA5 to complete its recognition by the viral S protein and prevent the attachment as well as having an inhibitory effect on endoribonuclease nsp15 (Khalil and Tazeddinova 2020; Elfiky 2021). Chlorogenic acid is a phenolic compound in propolis that hinders the binding of the S protein RBD domain and hACE2 at Gln325/Glu329, with smaller and more stable binding energy (Yu et al. 2020). Another chemical class, the flavonoids, has shown molecular dynamics activation to form flavonoid-protein complexes such as galangin, isorhamnetin, myricetin, hesperetin, and pinocembrin obtained from honey, propolis, pollen grain, and royal jelly. Moreover, molecular docking indicates a binding activity of galangin with S protein, contradicting viral entry (Jain et al. 2021). Isorhamnetin, a flavonoid, showed strong retention to hACE2 overexpression in HEK293 cells using chromatographical analysis. Hesperetin, myricetin and pinocembrin suppress the hACE2 with high binding energy to this target as well as inhibiting the cleavage activity of 3CL^pro^ (Berretta et al. 2020).

### Targeting viral replication

#### 3-Chymotrypsin-like main protease (3CL^pro^)

The literature review has revealed that natural compounds from non-plant sources block SARS-CoV-2 replication by suppressing 3CL^pro^ (M^pro^) activity within cell-free or cell-based assays. Among those inhibitors, the actions of different coumarins and naphthalene sesquiterpene derivatives isolated from marine sponges have been reported. Moreover, esculetin-4-carboxylic acid methyl ester and esculetin-4-carboxylic acid ethyl ester obtained from marine sponge, *Axinella cf. corrugata* (George & Wilson, Axinellidae) have shown an inhibitory effect on 3CL^pro^ with IC_50_= 46 μM in M^pro^ inhibition assays, an EC_50_ of 112 μM in Vero-cell assays (Coelho et al. 2020; Hamoda et al. 2021). An *in silico* study proved its interaction with M^pro^ as it showed high binding energy with (–8.42 kcal/mol) in addition to another naphthalene derivative, hamigeran b from marine sponge *Hamigera tarangaensis* (Bergquist & Fromont Hymedesmiidae) (–7.98 kcal/mol) (Vijayaraj et al. 2020). Chimyl alcohol (1-O-hexadecylglycerol extracted from *Desmapsamma anchorata* (Carter, Desmacididae) sponge and terpenoid (T3) from marine sponge *Cacospongia mycofijiensis* (Kakou, Crews & Bakus, Thorectidae) demonstrated suppressive effects on binding to M^pro^ in which the complex is stable and hydrogen bonds are involved during the complexation (Hamoda et al. 2021; Sepay et al. 2021).

Polycyclic guanidine alkaloids (PGAs) form one of the major groups of marine metabolites present in Poecilosclerida sponges such as Batzella, Crambe and Ptilocaulis, and some starfishes, such as *Fromia monilis* (Perrier, Goniasteridae) and *Celerina heffernani* (Livingstone, Goniasteridae). Docking, toxicity and molecular dynamic simulations reveal two promising candidates with low toxicity in the tested model. Rambescidin 786 has very good binding affinities against M^pro^ (–8.05 kcal/mol), nucleocapsid phosphoprotein (–6.49 kcal/mol), and nsp10 (–9.06 kcal/mol). Crambescidin 826 showed similar binding affinity against M^pro^ (–7.99 kcal/mol), in addition to S proteins (–6.95 kcal/mol), and nucleocapsid phosphoprotein (–8.01 kcal/mol). These actions are attributed to long ω-fatty acid chains which fit properly in the active sites (El-Demerdash et al. 2021).

Flavonoids obtained from honey, bee pollen, and royal jelly have been shown to be capable of inhibiting the activity of SARS-CoV M^pro^; among them are kaempferol and chrysin. It suppressed the 3a channel *in vitro* that is encoded by ORF 3a of SARS-CoV-2 and TMPRSS2 expression. In addition, the anti-inflammatory activity of kaempferol plays an essential role in fighting COVID-19 as it reduces interleukin-6 (IL-6), tumour necrosis factor alpha (TNF-α), and vascular endothelial growth factor (VEGF) via the ERK-NF-κB-cMyc-p21 pathway (Berretta et al. 2020). Chrysin interferes with S protein interaction with hACE1-2 as well as 3CL^pro^ via its binding to amino acid residues (SER-46, THR-24 and THR-26) of the main protease thus blocking both the replication of the virus and the emergence of the viral capsid protein. These two flavonoids not only affect viral pathogenesis, they also inhibit the inflammatory response as shown in an acute viral injury rat model and decreased fibrosis and pulmonary inflammation in an *in vivo* model. Chrysin has demonstrated an improvement in the NF-кB signalling pathway and modulation of neutrophil filtration in the oxidative stress dependent Nrf2 pathway (Abedi et al. 2021). In addition, it disturbs the viral invasion of host cells through its inhibition of RB of SARS-CoV-2 S protein (Khan and Siddiqui 2020). Rutin, and other honeybee and propolis compounds such as 3‐phenyllactic acid, caffeic acid phenethyl ester, lumichrome and apigenin are potential inhibitors of SARS‐CoV‐2 3CL^pro^ (Khalil and Tazeddinova 2020; Lima et al. 2021).

Cyclic dipeptides isolated from solid culture of fungi *Aspergillus versicolour* [(Vuill.) Tirab., Trichocomaceae] named indolyl diketopiperazines (IDPKs) have been characterized and screened virtually for their activities against SARS-CoV-2. Furthermore, aspamides A-B, aspamides F–G, brevianamide Q and brevianamide M have shown good docking scores as follow –5.389, –4.772, –5.146, –4.962, –5.158, respectively (Ding et al. 2020). Pseudotheonamides C and D are peptides isolated from the marine sponge *Theonella swinhoei* (Grey, Theonellidae), and are SARS-CoV-2 M^pro^ inhibitors. An initial docking analysis performed against 17 selected ligands was analysed by MD simulations. These two compounds were found to have a better energy score (–11.6 kcal/mol and –10.7 kcal/mol, respectively) than the comparative standard, Lopinavir HIV-1 protease inhibitors. The ligand-protein complex is stabilized by an enzyme interacting with phenyl groups occupied in small hydrophobic pockets of the enzyme (Gentile et al. 2020).

Other studies revealed that a phlorotannins isolated from edible brown algae *Ecklonia cava* (Kjellman, Lessoniaceae), named dieckol and eckol, exhibited the highest suppression rates of SARS-CoV 3CL^pro^ *trans*/*cis*-cleavage and the lowest binding energy to 3 CL^pro^ (–11.51 kcal/mol, –8.19 kcal/mol), respectively (Park et al. 2013; Arunkumar et al. 2021). One of the dieckol derivatives (DK70) has been proven to interfere with RBD–hACE2 interaction as it displayed good binding affinity with RBD. Furthermore, DK70 forms a stable complex with RBD due to the formation of hydrogen bonds, electrostatic and hydrophobic interactions, that are considered as residues mediating the hACE2–RBD interaction (Aatif et al. 2021). Trifucol is another phlorotannin obtained from the brown alga *Himanthalia elongata* ((Linnaeus) S.F. Grey,\Himanthaliaceae) and targets the S-glycoprotein (–7.5 kcal/mol) and 3CL^pro^ (–6.3 kcal/mol), respectively (Arunkumar et al. 2021). Different polysaccharides such as glycosaminoglycan from different marine species and bacteria, *Pseudomonas* sp., (Pseudomonadaceae) and laminarin from brown marine algae like *Laminaria digitata* ((Huds.) Lamouroux., Laminariaceae) are reported with M^pro^ higher binding energy at –7.98 kcal/mol and –7.81 kcal/mol, respectively (Vijayaraj et al. 2020; Arunkumar et al. 2021).

Phenolic compounds obtained from non-plant sources (honey, propolis and royal jelly) show inhibitory properties against PL^pro^, with quercetin and catechins among this category. Quercetin has shown to interfere with viral entry and replication and has better binding activity to PL^pro^, RdRp, 3CL^pro^, S protein and hACE2. *In vitro* assay has confirmed the ability of quercetin and quercetin-3-*O*-β-galactoside to restrict 3CL^pro^ with an IC_50_ value of 73 µM and 42.79 µM, respectively. The presence of hydroxyl group is essential for inhibitory action on 3CL^pro^ that has Gln189 as a crucial site of binding on 3CL^pro^. It blocks viral entry into Vero E6 cells (EC_50_ = 83.4 µM) and modulates the cellular unfolded protein response. It also reduces the inflammatory cascades by restraining cyclooxygenase-2 (COX-2) and iNOS, NF-κB, activator protein-1 (AP-1), and mitogen-activated protein kinase (MAPK) during infections. This results in suppression of the secretion of IL-1β, IL-6, interferon (IFN-γ), TNF-α, monocyte chemoattractant protein-1 and lipoxygenase (LOX). It demonstrates immuno-stimulatory effects by increasing the production of Th-1 derived IFN-γ and down-regulating Th-2 derived IL-4 (Khalil and Tazeddinova 2020). Catechin exhibits both anti-viral and anti-inflammatory properties via targeting pro-inflammatory IL-1β and IL-6 signalling pathways. It binds to the S1 ubiquitin-binding site of PL^pro^ and inhibits PL^pro^ leading to the abrogation of its deubiquitinase and deISGylation activity (Chourasia et al. 2021). By using a comprehensive computational approach, catechin was stabilized by the hydrophobic interactions with 3CL^pro^, cathepsin L, RBD of S protein, nsp6 and nucleocapsid protein with an energy range (–7.59 to –37.39 kcal/mol) (Mishra et al. 2021). *In vitro* results confirmed its inhibitory effects, with 85% inhibition of 3CL^pro^ (IC_50_ value of 73 ± 2 μM) (Mhatre et al. 2021). Both compounds act as ionophores for zinc ions that have beneficial anti-inflammatory effects in relation to COVID-19 (Agrawal et al. 2020).

Ilimaquinone is a terpene metabolite isolated from the marine sponge named *Hippospongia metachromia* (de Laubenfels, Spongiidae) that showed antiviral activity in an *in silico*, molecular interaction-based approach. Ilimaquinone exhibited molecular dynamics simulations of the PL^pro^–ilimaquinone complex with second highest binding energy towards PL^pro^ (–8.1 kcal/mol), nsp10 (–7.6 kcal/mol) and nsp14 (–8.1 kcal/mol) compared to remdesivir. Furthermore, 3CL^pro^ was also targeted by ilimaquinone with binding energy (–7.1 kcal/mol) similar to remdesivir and azithromycin as well as nsp16 having similar binding energy as ivermectin and remdesivir (Surti et al. 2020). Gallinamide A, a marine cyanobacterial depsipeptide, and some synthetic analogues have been reported to be powerful and selective inhibitors of cathepsin L, a lysosomal cysteine protease which helps coronaviruses to disperse their RNA content within cells. As would be expected, disabling this activity significantly reduces the severity of SARS-CoV-2 infection *in vitro* (White et al. 2021) and viral load has been shown to decrease with an IC_90_ of 88 nM in a SARS-CoV-2 viral infection assay (Ashhurst et al. 2020).

#### Other viral enzymes

Rutin is one of the flavonoids that affect enzymes such as helicase, RdRp, PL^pro^, and M^pro^ that play a major role in the viral replication cycle. It interacts via conventional H-bonds, π-cation π-alkyl π-sulfur and C-H bond interactions with high docking scores with –9.2 kcal/mol. It blocks hACE2 receptor virtually (–6.9 kcal/mol) and interferes with receptor binding with the viral S protein attachment. It suppresses the host toll-like receptors (TLRs) that mediate the inflammatory response in COVID-19 infection (Agrawal et al. 2020; Rahman et al. 2021). The inhibitory activity of limonin is shown through the high binding energy to RdRp and S protein from –9 to –7.1 kcal/mol (Berretta et al. 2020). Luteolin 7-rutinoside is also a flavonoid in propolis that binds efficiently to RdRp with nine H-bonds to the active site of RdRp (Alamri et al. 2020). a molecular docking study, gallic acid from honey showed higher binding affinity to the RdRp than the standard drug ribavirin, as well as showing significant pharmacokinetic activity (Abdo et al. 2021). Phycocyanobilins (PCBs) exist in different types of cyanobacteria, and, in algae rhodophytes, they show capability to inhibit RdRp, M^pro^ in *in silico* models. They have high binding affinity to RdRp (–7.2 kcal/mol), considerably higher than remdesivir, and they inhibit interaction with the RBD of S proteins through Vander Waal interactions (Geahchan et al. 2021).

### Anti-inflammatory and immunomodulation natural products

The enormous research effort in the fight against COVID-19 has presented a road map leading to much greater understanding of the disease as well as the agents that can manage its symptoms (Buszko et al. 2021). Two of the major hallmarks of COVID-19 are severe pneumonia and acute respiratory distress syndrome that occur as a result of post-viral activation leading to inflammatory response and destruction to the lungs, due to the heavy release of cytokines known as the cytokine storm (Hariharan et al. 2021). One of the striking features associated with disease progression and inflammatory response is the activation of the NF-κB pathway, leading to the induction of a variety of pro-inflammatory cytokines, such as IL-1, IL-2, IL-6, IL-10, TNF-α, IFN-γ-induced protein 10 and various chemokines. Also, immune response for COVID-19 cases includes the loss of plasmacytoid dendritic cells and basophils, with extreme T cell cytopenia, shown by the depletion of CD8+ T and γδT cell, even though T cells are not targeted by SARS-CoV-2. The involvement of inflammation and immunomodulation is highly emphasized, especially in treatment strategies as well as vaccines (Buszko et al. 2021).

Omega-3 fatty acids (FAs) are polyunsaturated fatty acids; specifically, eicosapentaenoic acid (EPA) and docosahexaenoic acid (DHA), that are present in abundance in fish oils and various algae, and which exist in the body in cooperation with the bi-phospholipid layer of the cell membrane of neutrophils to produce different mediators such as leukotrienes, that are known for their anti-inflammatory properties which may help to manage some of the complications of COVID-19. Thus, Omega-6 FAs may result in the stimulation of fewer inflammatory provoking mediators. Furthermore, a recent study showed that fish oil enhances the antiviral response via the inhibition of pro-inflammatory mediators such as IL-1β and IL-TNF-α, and protects the organism from COX and LOX pathways, and inhibits transendothelial migration of neutrophils and chemokine production. In addition, it modulates immune cell activation macrophages, neutrophils, T-cells, B-cells, dendritic cells, natural killer cells, mast cells, basophils, and eosinophils as well as their functions, such as inhibition of leukocyte chemotaxis and suppression of leukocyte-endothelial adhesive interactions, with a reduction of adhesion molecule expression. Recently, it was found that various subgroups of T cells such as CD4 cells, Th17 cells, and regulatory T cells become significantly more active in the presence of omega-3 FAs (Hathaway et al. 2020).

Plitidepsin (dehydrodidemnin B, 2) is a depsipeptide obtained from the tunicate *Aplidium albicans* (Milne Edwards, Polyclinidae) and is produced by PharmaMar under the name Aplidin^®^. It appears to hold greater potential than remdesivir in its activity against SARS-CoV-2, targeting the human protein eEF1A, which is essential for interaction with the N protein during the viral infection (Taglialatela-Scafati 2021). It has shown significant cytotoxic effects on the virus in human cells, with an IC_90_ of 0.88 nM. It is 27.5 nM more powerful than the remdesivir tested in the same cell line (White et al. 2021). It has been tested in different mouse models where it was initially administered before the mouse was infected with SARS-CoV-2, with the result that it significantly decreased viral load in a similar way to remdesivir. In the second model it suppressed lung inflammation more effectively than did the remdesivir, and this compound is now entering phase III clinical trials to test its effectiveness against COVID-19 (Taglialatela-Scafati 2021).

Melittin and apamin are one of the major compositions of bee venom *A. mellifera*. Melittin is recognized for its potential anti-inflammatory mechanism via decreasing the phosphorylation of an inhibitor of nuclear factor kappa B (IκB) known IkB kinase (IKK) as well as NF-κB. As consequence of IKK suppression, the secretion of TNF-α IL-1β and IL-6 is reduced with an inhibition of extracellular regulated protein kinases/p38 mitogen-activated protein (ERK/p38 MAP) kinase, leading to the inhibition of NF-κB pathway and matrix metalloproteinase-9 (MMP-9) expression and activity (Lin and Hsieh 2020). A nanoformulation of a combined complex of melittin and sitagliptin against SARS-CoV-2 virus revealed that expectations that the components would effectively pocket-accommodate SARS-CoV-2 3-CL^pro^ were fulfilled (Al-Rabia et al. 2021). Similarly, apamin decreases the NF-κB signal pathway in lipopolysaccharide-treated THP-1-derived macrophages as well as signal transducers and activators of transcription *in vitro*, thus decreasing the secretion of pro-inflammatory cytokines and Th2 lymphocyte chemokines (Gu et al. 2020).

Flavonoids and phenolic acids in honey products are recognized for their potential activity against inflammatory responses and several examples are mentioned here, such as kaempferol, chrysin, quercetin, catechin, syringic acid vanillic acid, luteolin. Syringic acid controls the inflammatory cells as well as inflammatory markers including IL-4, IL-5, IL-13, and TNF-α with its ability to suppress reactive oxygen species (ROS) and increase the anti-oxidant markers, to eases airway hyper-reactivity (Li et al. 2019). Vanillic acid suppresses neutrophil recruitment and its mechanism in controlling oxidative stress as well as inhibiting NFκB-related production of pro-inflammatory cytokine such as IL6, COX2, IL-1β and TNF-α. Luteolin disturbs lipopolysaccharide elicited inflammatory response and represses COX2, TNF-α, IL-6 and iNOS and ROS production inhibiting NF-κB and AP-1, MEK/ERK and PI3K/Akt pathways (Khalil and Tazeddinova 2020).

## Potential natural products alone or in combination in clinical data

The lack of an effective drug approved by the FDA as a first line treatment has forced many research groups to evaluate the clinical efficacy of different natural compounds. One of the fastest approaches is the repurposing of drugs previously approved by the FDA for COVID-19 therapy to create safe and potential regimens, such as hydroxychloroquine and remdesivir (Chakravarti et al. 2021). In preclinical and clinical studies, fucoidans have been proven to suppress inflammation and enhance innate immunity while decreasing inflammation and lessening the lung damage associated with acute respiratory viral infection (Fitton et al. 2020). Another combination has gone through clinical trials (NCT04425850) investigating Iota carrageenan nasal spray and Ivermectin oral drops for COVID-19 patients (ClinicalTrials 2021). A cross-sectional study was done of individuals affected by coronavirus, who used a combined therapy of melittin and apamin. This is a known traditional remedy containing bee venom that shortens the cellular infection period by 92% and enhances recovery from the infection by 97% (Caprazli and Kekeçoğlu 2021),

Due to its potential for effective anti-viral action, clinical trials for this flavonoid alone and in combination with others have been started. A phase 1 (NCT04452799) trial is under way to test hesperidin and diosmin in a hundred participants. Results have so far shown an improvement in host cellular immunity with anti-inflammatory activity leading to control of cytokine storms. Furthermore, this combination has shown protective effects against venous thromboembolism, which may prevent disease progression (Haggag et al. 2020). Quercetin phytosome, a lecithin-based delivery form of quercetin, was evaluated in a randomized, open-label, and controlled clinical study and resulted in shortening the duration for molecular testing to show negative, while at the same time weakening the severity of the symptoms (Di Pierro et al. 2021). Prophylaxis and treatment of COVID-19 using quercetin was clinically explored (NCT04377789) as a dietary supplement (ClinicalTrials 2021). A clinical trial by Pierro et al. revealed that quercetin is safe when combined with standard care and helps in improving the early symptoms of COVID-19 and in preventing their severity (Di Pierro et al. 2021). Gene analysis for quercetin combined with vitamin D has shown the alteration of 30% of genes encoding protein targets of SARS-CoV-2 as well as its interference with 85% of the viral proteins in human cells (Glinsky 2020). A quadruple treatment is also being investigated for the combined effects of zinc, quercetin, bromelain and vitamin C. Zinc functions as an adaptor of immune cell function, bromelain is an activator of natural killer cells and an anti-inflammatory agent, and vitamin C is an antioxidant as well as a mediator of collagen synthesis and immune regulation (Pal et al. 2021).

An ongoing phase II clinical trial (NCT04647604) is being conducted to test omega-3 supplementation as a possible treatment option in COVID-19 with minimal risks (Arnardottir et al. 2020). Another open-label, randomized, controlled study phase III (NCT04335032, NCT04836052) will be conducted where hospitalized patients take 2 g of EPA capsule and are observed for 28 days. Levels of oxygen saturation, mortality rate, hospitalization period and levels of inflammation will be measured (Rogero et al. 2020). Another trial will clinically investigate the effects of vitamin supplementation through randomized, double-blind, placebo-controlled trials of combinations of vitamins B, C and zinc, as well as omega-3, for the prevention and treatment of COVID-19 in which the infection rates, mortality and ICU admissions are measured (ClinicalTrials 2021).

## The possible relationships between the functional groups in chemical structure and viral proteins structure

A number of different chemical classes, flavonoids, peptides, terpenes and tannins, have demonstrated an ability to restrain different viral proteins due to their chemical diversity and the existence of different structural elements essential for their activities. These diverse and complicated structures of marine-derived compounds, along with their comparatively greater size, helps them to fit well into the target protein pocket. For example, phlorotannin isolated from edible brown algae such as dieckol and eckol share structural similarity and exhibit SARS-CoV 3CL^pro^ *trans*/*cis*-cleavage inhibitory effects with lowest binding energy. The eckol group of this marine phlorotannin is responsible for its binding with the 3CL^pro^. Furthermore, the presence of two eckol groups linked by a diphenyl ether in dieckol, results in strong binding inhibitory activity of SARS-CoV-2 3CL^pro^; this is because it is able to create strong hydrogen bonds with the catalytic dyad (Cys145 and His41) of SARS-CoV 3CL^pro^ and has a low binding energy (Park et al. 2013). Sulphated polysaccharides are a diverse group of natural compounds with different chemical structures. For example, sulphated and acetyl groups are reported to be successfully involved in the enhancement of antiviral activity. Moreover, the highest the degree of sulphation the best inhibitory activity against S protein interaction as indicated in SCSP which has shown the best inhibitory effect against pseudotype viruses by preventing virion attachment to the cells (Song et al. 2020).

Polyphenolics and flavonoids are classes with anti-viral and anti-inflammatory activities against COVID-19 infection. The presence of specific moieties, including hydroxy groups (–OH) and ketone groups (=O) in polyphenols, may affect interactions with amino acids within the target protein, such as S protein, hACE2, 3CL^pro^ and PL^pro^. Groups in ring C, including the carbonyl group at the C-4 position and 2,3-double bond, are important for the hydrogen bonding and the electrostatic interactions with the active site of 3CL^pro^ as shown in kaempferol and chrysin. In addition, the substitution of OH in C-3 with rhamnose or a rutinoside moiety in a flavonoid glycoside, as in rutin, results in a high binding affinity than aglycone. The rutinoside moiety affects the docking scores due the nature of the linked sugar with the skeletons and its position and may enhance the bioactivity of the compound. This moiety also adds stability to the protein complex compared to quercetin and isorhamnetin (Mouffouk et al. 2021). The presence of other moieties such as galloyl is required for its inhibitory activity on M^pro^ enzyme, as indicated by catechins, which have a galloyl moiety at C-3 in the C ring with higher M^pro^ inhibitory activity (Nguyen et al. 2021). Quercetin has better high binding affinities to the S protein in comparison to isorhamnetin that has a methoxy group in ring B causing steric hindrance and the absence of OH at C-3′ position reduces hydrogen bond formation, thus affecting the nature of the interactive residues with S proteins (Mouffouk et al. 2021). FAs are another important class of natural compound that could stabilize S protein’s closed conformation by binding to the fatty acid-binding pocket (FABP). When investigated, it has been reported that the varying chain length (16–24 carbon atoms), degree of unsaturation (1–6 conjugated double bonds) and the configuration of double bonds (*cis* and *trans*) have an impact on the inhibitory activity of omega-3 FAs. Furthermore, due to the existence of multiple double bonds, the hydrocarbon chain takes on the form of a coiled spring, and so the chance of steric clashing is reduced inside the restricted hydrophobic pocket. The double bond also enables advantageous π–π interaction with aromatic residues to occur inside the FABP. The position of the double bonds as well as chain length are important, since the longer the chain and the presence of ω-3 and ω-6 unsaturation, the higher the affinity to the S protein. As the long chain interacts with the highly hydrophobic FABP in one subunit, the other ionized carboxylate group binds to Arg 403, Arg 408, and potentially with Lys 417 in the other subunit via hydrogen bonding and electrostatic interactions, to close the RBD and limit the access to hACE2 receptor (Vivar-Sierra et al. 2021). It can be seen that the ability of the various chemical classes of mentioned natural products to bind to target proteins is due to the diversity of biological activities that are possible within these groups. Using computer modelling we can forecast the types and strength of interactions between ligands and targets, which helps to identify which leads are worth following in the search for potential drugs.

## Conclusions and future prospect

In the face of this enormous global challenge, searching of a specific drug for the treatment of COVID-19 is continuous, which has caused unprecedented disaster in many areas of life, not only in terms of health, but also socially and economically. The entire scientific community is working tirelessly to find and develop a drug therapy for COVID-19 that is safe and effective. The low toxicity, availability, and sustainability of natural products may be key factors in the development of successful, naturally-derived drugs for COVID-19, especially as there are many such drugs that have already been developed and are used for antiviral and anti-inflammatory purposes.

In this review, we have summarized pre-clinical and clinical data available from recent research that investigates natural products from marine, microorganism and animal sources within the context of COVID-19. Most of the published literature on active natural compounds has identified functional groups such as flavonoids, polyphenols, peptides, terpenes and tannins, which have inhibitory properties including antiviral, anti-inflammatory and immunomodulatory activity against various viral and host proteins including 3CL^pro^, PL^pro^, S, hACE2, NF-κB and TNF-α. As reviewed in this paper, there are registered clinical trials taking place to investigate naturally-derived products such as Iota carrageenan nasal spray and Ivermectin oral drops (NCT04425850), EPA capsules (in study phase III) (NCT04335032, NCT04836052) and omega-3 supplementation (NCT04647604) for use as possible treatment options for COVID-19 with minimal risks. The results so far are showing improvements in oxygen saturation, lower mortality rates and hospitalization periods and lower levels of inflammation and higher innate immunity. Furthermore, exploration of the synergistic effects of logical combinations of compounds with different mechanisms of action, such as hesperidin and diosmin, as well as a quadruple treatment of zinc, quercetin, bromelain and vitamin C, show enhanced anti-inflammatory and immunomodulatory effects.

Although published clinical data exists for the reviewed compounds, one of the challenges is that much of today’s research is either theoretical and carried out *in vitro*, or it has yet to be validated analytically, and therefore we still have a long way to go in terms of drug development including biological analysis and optimized extraction and production. Another challenge is the emergence of SARS-CoV-2 variants, α, β, γ and δ, and although there are many vaccines gaining WHO EUL/PQ authorization such as Pfizer, Moderna and AstraZeneca, their effectiveness against the variants is still under investigation. Thus, researching potential drugs from natural sources could prove a promising way to treat the symptoms and effects of COVID-19.
